# From Reductionism to Reintegration: Solving society’s most pressing problems requires building bridges between data types across the life sciences

**DOI:** 10.1371/journal.pbio.3001129

**Published:** 2021-03-26

**Authors:** Anne E. Thessen, Paul Bogdan, David J. Patterson, Theresa M. Casey, César Hinojo-Hinojo, Orlando de Lange, Melissa A. Haendel

**Affiliations:** 1 Department of Environmental and Molecular Toxicology, Oregon State University, Corvallis, Oregon, United States of America; 2 Ming Hsieh Department of Electrical and Computer Engineering, Viterbi School of Engineering, University of Southern California, Los Angeles, California, United States of America; 3 University of Sydney, Sydney, Australia; 4 Department of Animal Sciences, Purdue University, West Lafayette, Indiana, United States of America; 5 Department of Earth System Science, University of California, Irvine, California, United States of America; 6 Department of Electrical Engineering, University of Washington, Seattle, Washington, United States of America

## Abstract

Decades of reductionist approaches in biology have achieved spectacular progress, but the proliferation of subdisciplines, each with its own technical and social practices regarding data, impedes the growth of the multidisciplinary and interdisciplinary approaches now needed to address pressing societal challenges. Data integration is key to a reintegrated biology able to address global issues such as climate change, biodiversity loss, and sustainable ecosystem management. We identify major challenges to data integration and present a vision for a “Data as a Service”-oriented architecture to promote reuse of data for discovery. The proposed architecture includes standards development, new tools and services, and strategies for career-development and sustainability.

## Abstract 2

Data integration is key to the reintegration of biology and the pursuit of global issues such as climate change, biodiversity loss, and sustainable ecosystem management. This Essay defines the primary challenges in data integration and presents a vision for a "Data as a Service" (DaaS) oriented architecture that enables frictionless data reuse, hypothesis testing, and discovery.

## Introduction

Life on Earth is an interplay of interacting biological systems and geological processes that evolved over approximately 3 billion years and is represented by more than 2 million extant species. It is this complex system that creates the context for efforts to maintain global biodiversity while ensuring the health and well-being of our growing human population. Progress will require input from many disciplines to understand and manage our challenges [1]. Decades of reductionist research have led to extraordinary insights but have produced many subdiscipines with differing technical and social practices. If we are to solve societal problems, we must gain access to and bring together data from many disciplines. This is not straightforward because of the heterogeneity of data and associated conventions among communities. One clear and present challenge is how best to integrate data from the subdisciplines.

**Fig 1:**
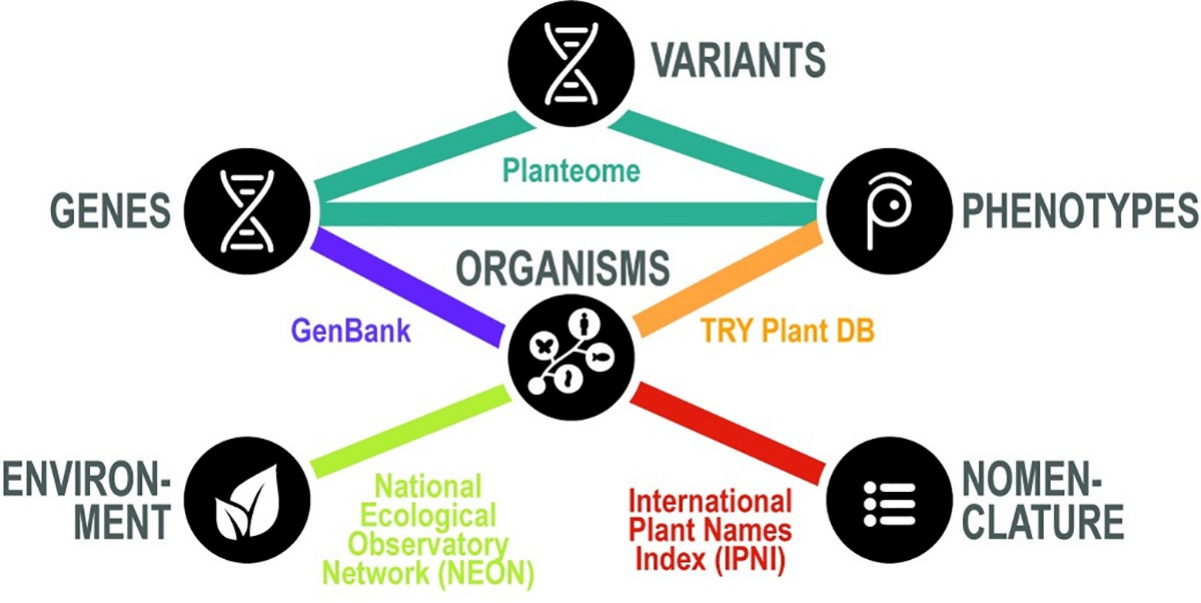
Reintegrating data to understand phenotype. Most biological data repositories only cover one part of the biological picture and must be integrated with other repositories in order to see the whole. Understanding phenotype requires data about genes, gene variants, organisms, environments, and taxonomy with nomenclature. Using plant phenotypes as an example, a minimum of 5 repositories are required to hold and curate relevant information. The repositories listed are only examples and do not represent all available resources.

Open access to data has the potential to democratize innovation by making it easier for third parties to reuse data and test solutions to complex problems that subdisciplines cannot address alone [2], but open access is merely a prerequisite. An important example of one of these complex problems is understanding the effect of genes and environments on observable phenotypes. Understanding phenotypes requires data about genes, variants, organisms, and environments, among others, and much of these data are open but not truly integrated (Fig 1). Understanding complex phenomena, such as expression of phenotypes, requires access to integrated data.

Our current limited ability to integrate data across scale, methodologies, and disciplines [3] impairs progress with multiscale, heterogeneous, non-Gaussian, and non-Markovian networks of dynamical systems [4]. Aligned with the vision of the National Science Foundation, we advocate for a comprehensive approach to data integration for predictive modelling of complex systems. Underlying issues of data sharing, integration, and reuse (Box 1) have been discussed widely [2,5–23]. Solutions to impediments have been proposed and variously implemented in the context of Data as a Service (DaaS). We use this term to point to evolving service-oriented infrastructures which provide data for third-party reuse. The infrastructure acquires data from primary sources and delivers fit-for-purpose content through trusted and curated repositories, inclusive of commercial and noncommercial agencies. DaaS aims not to serve a particular research agenda but is agile and adaptive such that it can support any project. The infrastructure is characterized by best practices, globally accepted standards, and is supported by a community of data experts, all of whom receive credit for their contributions [24]. One example of DaaS-oriented infrastructure for biology is CyVerse. Yet, challenges persist with increasing the scale and scope of data types that can be integrated and provided via DaaS, and with incentives for making data persistently available for use by third parties. Calls from high-profile scientific organizations [25,26] for unification of biological data are driven by improved computing power, new computational methods, maturing data standards, emerging exploratory protocols, advances in collaborative environments, changing attitudes about data sharing, and trustworthy data curation.

**Box 1. Data integration challenges**

Challenges in the nature of the data

- Data are highly variable;
- Data are collected on multiple spatiotemporal scales;
- Data generation has gaps;
- Data are not discoverable.

Challenges in the nature of biological systems

- Large biological systems are highly variable and dynamic;
- Biological systems do not comply with simple statistical models.

Challenges in the nature of data infrastructure

- The data infrastructure does not incentivize sharing;
- The data infrastructure is difficult to establish and sustain;
- Use of the data infrastructure requires specialized training;
- The data infrastructure may have restrictive licensing.

**Fig 2:**
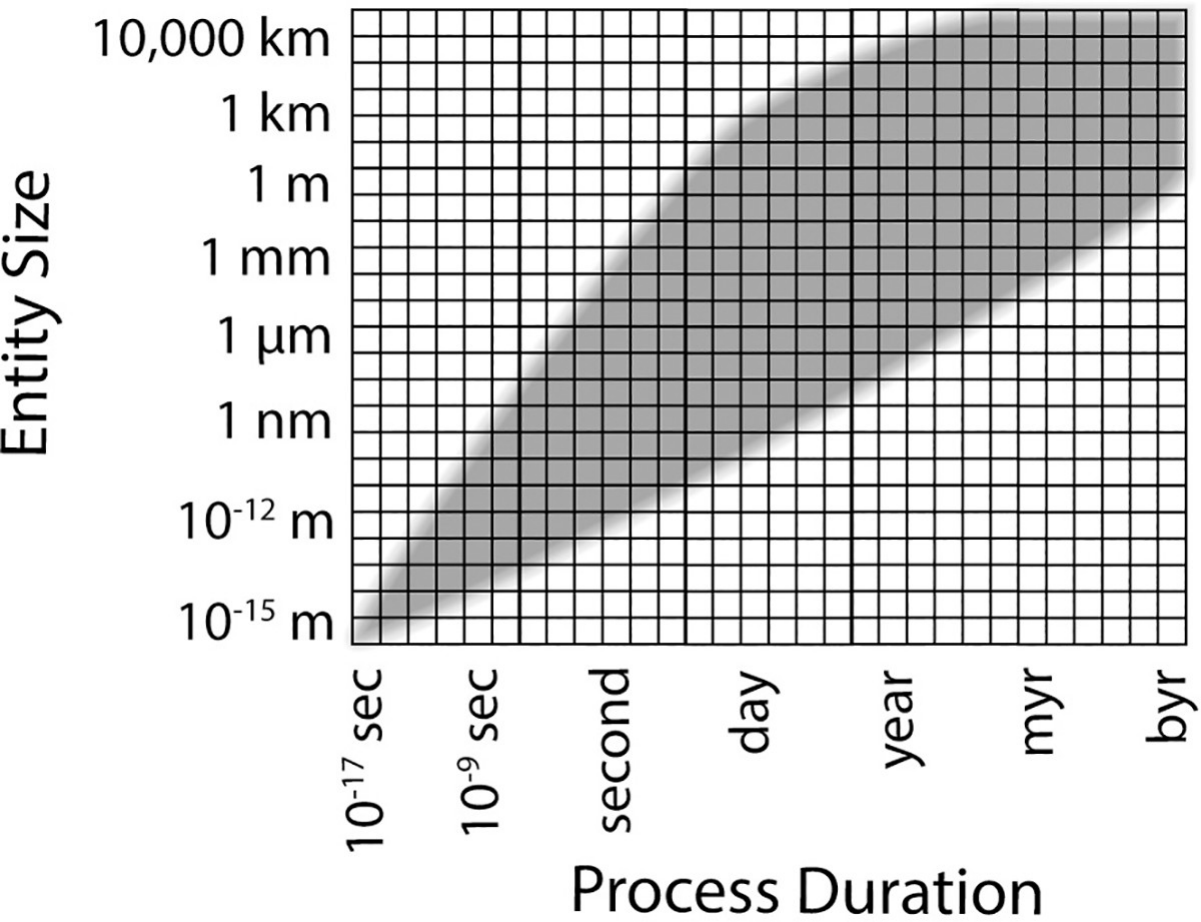
Envelope of life. Life sciences study entities (vertical axis) and processes (horizontal axis) that occur across a broad range of (logarithmic) scales. The shaded area emphasizes where biologically relevant processes occur.

We advocate for a DaaS-informed strategy to build bridges between data types. Data-centered collaborations that are aware of the full scope of biology (Fig 2) will lead to novel cyberinfrastructures that enable cross-disciplinary data integration. A new cyberinfrastructure will enable currently unimagined interdisciplinary investigations and promote paradigm shifts in biological research [27]. Building on previous reviews [28], we summarize outstanding barriers to effective data practices (Box 1) and make proposals that will help us overcome barriers.

## Foundational infrastructure components

The development of a service-oriented DaaS architecture with appropriate human expertise and technical infrastructure will improve integration of currently separated data and enable transdisciplinary collaboration. The idea of access to data on demand across a broad front is not new. Several repositories and aggregators provide biological data on demand (e.g., [29–32]). We advocate for extending DaaS infrastructure to address persistent barriers to data sharing and integration. Below, we outline 7 challenges and propose opportunities to resolve each of them, which we refer to as foundational components.

### Licensing of data and software

The open science movement rests on a foundation that research data, software code, and experimental methods should be publicly available and transparent unless privacy or confidentiality is an issue [8]. *The first foundational component of DaaS is straightforward*, *permissive*, *human- and machine-comprehensible licensing*. Licenses need to be simple to reduce confusion [33–36] and designed to allow automated access to data. A call to license data and software is not new, but licensing, copyright, and data use agreements are poorly understood [33,37,38], delaying their application. A restrictive license, in this context, is any license that places additional requirements or caveats on use of the data. Investment in data citation, data publication, microannotation, and nanopublication [39–44] will reduce the need for the restrictive licenses and nonstandard use agreements that are often in place to track reuse and impact. A global system of interconnected data with automated methods of tracking use and apportioning credit requires standardized, machine-readable licensing and data use agreements.

### Data integration is still a largely manual task

As with people and ideas, data have been siloed within discipline-specific, project-specific, or lab-specific environments. The key to integrated data among silos is the universal adoption of community-developed standards [45] (Box 2). Even with most current standards, substantial manual effort can be required to map overlapping schemas or transform data from one context to another. Standards which describe data and metadata, document formats, content, protocols, and vocabularies are essential for automated integration [46]. With appropriate standards, the transformation workflow can be automated, it can include multiple quality checks, perform transformations, promote reproducibility, identify provenance, reduce manual errors and inconsistencies; all at a lower cost. The automation of integration will add machine-actionable links across repositories that will result in a network of data repositories. An example that integrates across repositories is Biolink, a graph-oriented data model that was developed for a biomedical use case but is being extended to the rest of biology [47]. Domain standards are a concrete way to increase interoperability and integration across repositories given that the basic elements (i.e., semantic types) of biology and the relationships between them are identified consistently across resources. Yet, the context-dependent nature of data transformation is not well represented by existing standards. A solution may lie with microschemas, highly localized data models to ensure accurate context-dependent data transformation similar to the GA4GH schemablocks concept [48] that can facilitate automation of highly contextual data like total cholesterol or ocean acidification.

**Box 2. Standards development and governance**

Successful standards require a sustained, iterative process of continued development that allows for changes to the standards, to metadata, and to the data they describe. Community-driven, consensus-building approaches, rather than an exclusively top-down approach, allow each community of practice to develop their own standards. This process is typically codified within a governance document that describes how to update the standard, resolve disputes, manage documents and versions, etc. Effective governance, including a Code of Conduct, can make a big difference in whether or not members feel welcome and effective, which drives participation. Governance should be well documented, community-driven, and reviewed at intervals that are sensible for the degree of change in the data and methods being standardized. The bottom-up development of sustainable, useful standards for data aggregation and integration necessitates a robust governance process that can represent community buy-in and provide a handbook for collaboration.

The second foundational component of DaaS is standards to support machine actionable metadata and corresponding algorithms for automated standardization and integration.

Automated data integration requires standards. The incentive structures for most academics do not include the development of standards or scientific software as intellectual outputs. A lack of data standards impedes progress, and it is now timely to acknowledge efforts to improve standards and the tools and services which support their use [15,49].

### Metadata are underappreciated

Despite the importance of metadata, their creation is still neglected [50]. Collecting metadata at the same time as data they describe is a recognized best practice [9], but this does not always happen and substantial amounts of data have been collected without standard metadata. *The third foundational component of DaaS is algorithms for automated metadata creation and standardization with documentation and provenance*. New tools are needed to automate the generation of metadata across data types and scales, where possible [46]. Machine learning (ML) and artificial intelligence (AI) can enhance metadata with standards and detect appropriate protocols for data normalization. High-priority automated tasks include named entity recognition, data and semantic typing, and protocol detection. Algorithms for semiautomated crowdsourced curation will benefit quality control and other tasks that cannot be fully automated [51]. Some entity recognition algorithms already exist [52–54] but have not yet received wide adoption because of problems with usability, sustainability, and discoverability of the tools; or because of the need of changes to work practices. Without a strong user community, it will be hard to recruit resources to create and improve these tools. One perception is that metadata preparation and documentation is altruistic and without significant impact [19,50]. While not a universal view [23,55], better professional rewards that value metadata creation and associated tools are needed.

### The quality of data and metadata is variable

Issues relating to quality include social and technical aspects of the data, metadata, data providers, aggregators, and repositories [15,56–59]. Numerous studies explore trust in data sets and the expectations of users [15,42,43,60,61], but there is no widely adopted, formal process for judging data set quality. The peer review system for publications, even with its flaws [62], can provide a starting point for an assessment of the quality of data sets [42]. *The fourth foundational component of DaaS is a simple*, *predictable*, *and transparent system for peer review of data*. While some repositories have a review process for submitted data sets that may include automated checks, and data publication journals can review data set documentation, routine rigorous peer review of data has not been implemented. One deterrent is that the pool of likely reviewers is already overburdened. If peer review of data sets is to have any hope of implementation, an infrastructure that puts reviews to good use and apportions credit for conducting reviews is needed. A supplementary approach is to use annotation technology to enable feedback on data sets and data atoms by end users [63].

### Data use and contributions are hard to track

Researchers typically use citation metrics of publications as a measure of the impact of their career. The other types of activity, such contributions to an infrastructure, or third-party reuse of data, are often neglected because, historically, no comparable systems exist to track other endeavors. DataCite [40], an advocate for data access and discovery, developed a data set citation standard with DOI assignments that is used in several disciplines, but additional supporting infrastructure is needed to fully understand what a data citation, and the resulting metrics, means for the career of the producer and the value of the data themselves [64]. A roadmap for a system that supports standardized data-level metrics [65] was developed by MakeDataCount.org and is available for implementation. This roadmap fills many of the important technical gaps but requires resources to increase adoption across repositories, publishers, institutions, and researchers in order to create a system of data metrics comparable to publication metrics.

The mutable nature of data sets raises issues with identifiers and versioning that do not apply to publications [43]. For reproducibility, a published analysis must point to a persistent version of the data that were used, even if a small change was made after publication. In addition, credit needs to be apportioned appropriately when data sets are curated, subdivided, combined, vetted, and upgraded in ways that manuscripts are not. This raises several provenance and attribution issues that can only be addressed by well-documented versioning with a robust chain of provenance (for an example system, see [66]). When a data set is downloaded for analysis, that chain is usually broken, making it nearly impossible to communicate usage metrics back to the source and other agents in the data supply chain. Mechanisms and infrastructure that bring analyses to the data will better reveal the entire workflow so that it can be reproduced and refined. *The fifth foundational component of DaaS is a transparent pathway for preserving provenance and attribution within analytical environments*. Many computing environments for large data sets comply with this component because most researchers do not have the local resources to manipulate very large data sets. Researchers with small data sets that can be handled by spreadsheets are less likely to preserve these metadata. Collaborative environments with a support infrastructure similar to git, GitHub, or FilteredPush [63] engage all participants in data stewardship and to make the pathway of content flow, value-adding, and analysis visible.

### Good data managers and curators are scarce

If we are to make full use of rapidly changing technology, we need data expertise coupled with in-depth biological knowledge [67]. People with such skills are rare. Increasingly, biologists will require data training, but this is not sufficient to create new advanced tools. Rather, we require a professional development structure for a community of biologists with advanced expertise in data management, curatorship, software development, and information science. *The sixth foundational component of DaaS is a method that attributes and credits the work of professional data managers and curators that is equal to manuscript citation and can accommodate microannotation and nanopublication*. The Contributor Attribution Model (CAM) [68] used in combination with microannotation or nanopublication [39,69,70], wherein metadata are associated with individual data atoms (smallest usable elements), can underpin a system of attribution tracking where individual credit cascades through the long pathway of content flow [37,39]. CAM builds on the work of groups like CASRAI [71] by using CRediT [72] to inform the Contributor Role Ontology—the source of role terms in CAM [73]. (Domain-specific groups like CASRAI are an integral part of developing the community standards discussed above.) There are a few existing systems for recording attribution such as ORCID [74], OpenVIVO [75], and rescognito [76] that have begun to tackle the issue of credit for data work. With more transparency, the investment in making data more reusable becomes more measurable and removes the disincentive of working hard for no reward [19,22,60].

### Sustainability for data remains elusive

The current strategy for funding scientific research leaves most data unsupported after completion of the project. An essential but often overlooked aspect of data integration is long-term preservation. Repositories, including museums and libraries, have the knowledge and expertise to provide sustainable preservation of data [27], but many data repositories accommodate only a single subdiscipline or data type (with a few exceptions, e.g., [29]). CoreTrustSeal promotes best practices for repositories committed to the long-term preservation and curation of data [77].

Fitness of data can corrode with time, and this requires maintenance of the schemas, metadata, and even data (Box 2). An example is when names of and concepts for taxa change [37,56], but their use as metadata remains uncorrected. While many repositories regularly update their content, including the creation of new data products (e.g., [78]), others lack the resources or disciplinary skills to make these updates quickly. This leads to dissatisfaction with the current ecosystem of long-term data support [15,56]. One reaction is for researchers to maintain data locally; but the probability that project-oriented data environments are available for reuse decreases by 17% per year [79]. *The seventh foundational component of DaaS is low-cost*, *reproducible workflows that convey data and metadata from creation to an accessible trusted repository that delivers data that are fit for purpose in perpetuity*. Lack of preservation resources places much of our collective digital knowledge in jeopardy, is dismissive of the investment in creating data, threatens future insights that might be drawn from data, and decreases our ability to engage in reproducible science.

### Our vision of a reintegrating biology

It is inevitable that an extensive integrated data environment will emerge. With it will come new opportunities for discovery, devices to address problems with greater scale and scope, and the quality of insights will improve [80]. There are several leaders in this developing space, including CyVerse—an open science workspace for collaborative data-driven discovery that offers an infrastructure that supports data throughout its life cycle regardless of platform [81]. CyVerse supports access to high-performance computing and the packaging of data and software together in reusable “Research Objects” such as those used in the Whole Tale Environment [82]. A DaaS model can promote the emergence of a more extensive network of curated, interlinked data within an environment that is rich in tools and interoperable services. Progress is impeded because much of the required self-reinforcing infrastructure is absent. We emphasize 2 barriers to achieving the foundational components discussed here. First: motivating the sustained community participation that is needed to develop and implement discipline-specific data integration solutions—especially in respect of discipline-specific and domain-specific standards that enable the automated components that make large-scale integration tractible. Second: the data citation and peer review infrastructure (beyond DOIs) needed to motivate professional participation in data-centric activities does not yet exist. The interconnected nature of these problems means that partial solutions, which are easier to fund, will not have the desired impact. The role of publishers and aggregators of intellectual output, like ORCID, in making this vision a reality cannot be overstated [83,84]. Some of the early progress with incentivizing data sharing were led by the requirements of publishers [85], and they remain a major driver of data sharing behaviors [22]. Publishers, researchers, and repositories will need to collaborate to adopt and enforce a standard of data citation and peer review that, combined with infrastructure supporting provenance, microattribution, annotation, and versioning proposed here, can perpetuate credit across a workflow. Well-formed attribution metadata can make essential data-related tasks just as visible as traditional publications. This is key to improving the academic incentive structure that currently demotivates investment in data-centric research practices.

## Summary

Our ability to address issues that draw on many subdisciplines of biology will improve with integrated access to data across diverse types. Our vision is that disciplinary boundaries will break down to reveal a virtual pool of integrated data from many subdisciplines. This pool of data will need to be supported with an ecosystem of automated management processes, bridging metamodels, services, and semantic technologies. A DaaS approach can lead to decentralized repositories where knowledge and contributions are part of a distributed and shared global network that maintains provenance and attribution for all participants. To overcome impediments, we propose that the following 7 components will foster DaaS in biology:

- *Straightforward*, *permissive*, *human- and machine-comprehensible licensing;*
- Standards to support machine actionable metadata;
- Algorithms that automate the creation of metadata where possible;
- *A simple*, *predictable*, *and transparent system for peer review of data;*
- A transparent pathway for preserving provenance and attribution within analytical environments;
- A method for attributing and crediting the work of data managers and curators;
- *Low-cost*, *reproducible workflows that support the movement of data and metadata from creation to trusted repositories of data that are fit for purpose*.

Advances in automated data management practices, community standards, and data publication infrastructure put these components within reach. Investments in data infrastructure will increase data usability, impact, and marketability of data through a DaaS model and a shift in professional incentives that values investment in this area. Addressing these challenges will lead to an improved basis to answer current big questions in biology and contribute science-based solutions to the most pressing social and environmental problems.
